# Type of Wheelchair Used before Nursing Home Admission and Fall-Related Fractures after Nursing Home Admission

**DOI:** 10.31662/jmaj.2025-0194

**Published:** 2025-09-19

**Authors:** Ai Suzuki, Kazuaki Uda, Taeko Watanabe, Nanako Tamiya

**Affiliations:** 1 Doctoral Program in Public Health, Department of Health Services Research, University of Tsukuba, Ibaraki, Japan; 2 Health Department, Tsukuba, Ibaraki, Japan; 3 Department of Health Services Research, Institute of Medicine, University of Tsukuba, Ibaraki, Japan; 4 Health Services Research and Development Center, University of Tsukuba, Ibaraki, Japan; 5 Department of Social Science, Center for Gerontology and Social Science, National Center for Geriatrics and Gerontology, Aichi, Japan

**Keywords:** fall-related fracture, long-term care, nursing home, Japan, wheelchair

## Abstract

**Introduction:**

Residents who use multifunctional wheelchairs before nursing home admission often face discontinuation of such devices after nursing home admission. However, basic data on this issue remain limited. Therefore, in this study, we aimed to provide preliminary data by investigating the occurrence of fall-related fractures after nursing home admission, stratified by the type of wheelchair used before nursing home admission.

**Methods:**

We used linked long-term care claims data, care-need certification survey data, and medical claims data from Ibaraki Prefecture, Japan. Participants were older adults who were newly admitted to nursing homes directly from home between July 2018 and February 2019 and had used wheelchair rental services before nursing home admission. We divided the wheelchairs into two types using long-term care claims data: multifunctional and standard wheelchairs. The outcomes of interest were newly occurring fall-related fractures within the first month of nursing home admission, identified from medical claims data. Fracture incidence was compared between the two wheelchair types, and information of residents with fracture were described.

**Results:**

The study included 215 participants. The participants’ median age was 88 years (interquartile range, 83-93); 153 (71.2%) were female, and 96 (44.7%) used a multifunctional wheelchair before nursing home admission. There are no fall-related fractures occurred among standard wheelchair users, whereas five fractures were observed among multifunctional wheelchair users.

**Conclusions:**

Residents who used multifunctional wheelchairs experienced more fall-related fractures after admission than those who used standard wheelchairs before nursing home admission. Further research is needed to better understand the issue of service continuity by investigating wheelchair use after nursing home admission and the circumstances of fall-related fractures.

## Introduction

In Japan, the proportion of older adults aged ≥65 years was 29.1% in 2023 and is projected to reach 38.7% by 2070 ^(1)^. Consequently, the number of older adults residing in long-term care facilities has been increasing ^(2)^. Under the Japanese long-term care insurance system, there are three types of long-term care facilities: nursing homes (referred to as *kaigoroujinhukushishisetsu* or *tokubetsuyougoroujinhoumu* in Japanese), geriatric intermediate care facilities (*kaigoroujinhokenshisetsu*), and medical long-term care sanatoriums (*kaigoiryoin*) ^(3)^. Among these facilities, nursing homes accommodate the largest number of residents ^(2)^. Because nursing homes primarily serve older adults with severe disabilities, more than half of residents rely on wheelchairs in their daily lives ^(4)^, with most wheelchair users spending 7-9 hours/day on the wheelchair ^(5), (6)^.

In April 2000, with the implementation of the long-term care insurance system, community-dwelling older adults gained access to wheelchairs equipped with functions tailored to their physical needs through assistive device rental services. For example, those who need special functions to maintain proper posture in a wheelchair owing to conditions such as kyphosis or muscle weakness can rent wheelchairs equipped with functions such as back support frames designed to accommodate kyphosis or lateral trunk support, and those whose body dimensions do not fit standard wheelchairs can rent wheelchairs equipped with functions such as adjustable seat width, height, and frame angle. Under this system, most users rent wheelchairs for a 10% co-payment, whereas older adults with a high income pay a 20% or 30% co-payment ^(7)^.

On the other hand, older adults residing in nursing homes are not eligible for assistive device rental services and must rely on facility-owned wheelchairs. Most of the facility-owned wheelchairs are nonadjustable standard wheelchairs with sling seats that do not take into account the body dimensions and physical functions ^(8)^. Therefore, if nursing home residents had used a wheelchair with various features to suit their physical functions rather than a standard wheelchair before nursing home admission, they may not be able to use a similar wheelchair after nursing home admission. The use of unsuitable wheelchairs can lead to adverse events, such as falls from the wheelchair ^(9)^, resulting in fractures. Notably, fractures should be prevented because they increase the risk of mortality ^(10), (11), (12)^.

In summary, under the long-term care insurance system, because nursing home residents are not eligible to use rental services, residents who used wheelchairs with various functions before nursing home admission may not have access to similar wheelchairs after nursing home admission, which could lead to fall-related fractures. However, basic data, such as the type of wheelchair used before nursing home admission and how many wheelchair users experience fall-related fractures after admission, remain limited. Therefore, in this study, we aimed to provide preliminary descriptive data by investigating (1) the occurrence of fall-related fractures after nursing home admission by type of wheelchair used before admission and (2) characteristics of residents with fall-related fractures.

## Materials and Methods

### Study design and data source

This was a descriptive study that used retrospective cohort data. We used long-term care claims data and care-need certification survey data from the long-term care insurance system and medical claims data from the National Health Insurance and Latter-stage Elderly Medical Care System in Ibaraki Prefecture, Japan, covering the period from April 2018 to March 2019. Long-term care insurance provides services for the persons aged ≥65 years who need long-term care and for those aged 40-64 years with specific geriatric diseases ^(7)^. National Health Insurance covers self-employed and unemployed persons aged <75 years ^(13)^, whereas the Latter-stage Elderly Medical Care System covers persons aged ≥75 years and certain disabled persons aged ≥65 years ^(14)^. The long-term care claims data include information regarding the long-term care services provided and basic information of long-term care residents, such as sex, age, and care-need levels, and the care-need certification survey data include information regarding physical and mental functions of residents. The medical claims data include information regarding medical diagnoses and prescription medications. We obtained anonymously linked data from the government of Ibaraki Prefecture. Informed consent from individuals was waived because of the anonymous nature of the data.

### Study population

The study enrolled individuals who met the following criteria: (1) aged ≥65 years, (2) newly admitted to a nursing home directly from their home setting between July 2018 and February 2019, and (3) used a wheelchair rental service before nursing home admission. Individuals were excluded if they were unsure of the type of wheelchair they had used before nursing home admission or if their long-term care claims data and medical claim data were not linked owing to key identification issues.

### Type of wheelchair

The long-term care claims data include a code for each wheelchair device (Technical Aids Information System [TAIS] code) ^(15)^. Wheelchair devices were identified using this TAIS code and divided into two types based on their functions: standard and multifunctional wheelchair (Table 1).

**Table 1.** Type of Wheelchair.

Wheelchair type	Multifunctional wheelchair		Standard wheelchair	
	Self-propelled or caregiver-assisted wheelchair	Tilt or reclining wheelchairs	Self-propelled or caregiver-assisted wheelchair	Tilt or reclining wheelchairs
Function	・Adjustable structure		・Sling seat and back support	
	・Low seat height (≤35 cm)		・Sling seat and back support with adjustable tension.	
	・Back support frames designed for kyphosis		・Removable arm support	
	・Lateral support		・Removable leg support	
	・Automatic braking to prevent backward movement	

Multifunctional wheelchairs have various functions, such as an adjustable structure, low seat height (≤35 cm), and back support frames designed to accommodate kyphosis, whereas standard wheelchair models have standard functions, such as a sling seat and sling back support and removable arm or leg supports. Both types include self-propelled or caregiver-assisted models with convertible seating positions, such as tilt wheelchairs (where the entire seat pan and back support unit incline) and reclining wheelchairs (where only the back support reclines).

### Outcome

The outcome of interest was newly occurring fall-related fractures within the first month of nursing home admission. Referring to previous studies, fall-related fractures were defined as fractures of the hip, forearm, and humerus ^(12), (16)^. Each fracture was identified using the International Statistical Classification of Diseases and Related Health Problems, 10th Revision (ICD-10) codes from medical claim data, and procedure codes for surgery (e.g., open reduction internal fixation), cast immobilization, and imaging examination (e.g., radiography). The corresponding ICD-10 codes were as follows: hip fracture (S72.0, S72.1, S72.2), forearm fracture (S52), and humerus fracture (S42.2, S42.3, S42.4). The corresponding procedure codes were as follows: surgery (K044, K045, K046, K046-2, K046-3, K073, K073-2), cast immobilization (J122), and imaging examination (E001, E002, E003). Newly occurring fall-related fractures were defined as fractures that occurred without a diagnosis of the same fracture within the 90 days before their fall-related fractures occurrence.

### Participants’ characteristics

We obtained the participants’ age, sex, care-need levels, comorbidities, prescribed medications, and physical function (sitting, standing up, transferring). Comorbidities and medication were identified using medical claims data, and physical function was identified using care-need certification survey data. The corresponding ICD-10 codes for comorbidities were as follows: dementia (F00-F03, G30), cerebrovascular diseases (I60-I69), visual deficits (H53, H54), and depression (F32, F33) ^(17)^. Similarly, the prescribed medications were identified using the Anatomical Therapeutic Chemical Classification System codes: antipsychotics (N05A), benzodiazepines (N03AE, N05BA, N05CD), antidepressants (N06A), sedatives and hypnotics (N05C), and antihypertensives (C02, C03, C07, C08, and C09) ^(18), (19)^.

### Statistical analysis

First, we described the participants’ characteristics according to the wheelchair type used. We compared the characteristics between the groups using the Student’s *t* test for continuous variables, chi-square test for binary variables, and Wilcoxon rank sum test for ordinal variables. Next, we compared the number of fall-related fractures for each wheelchair type. Finally, we described information on residents with fall-related fractures, including fracture site, the type of wheelchair used before nursing home admission, and physical function.

Data management and analyses were conducted using Stata 17 software (StataCorp, College Station, TX, USA). The p values <0.05 were considered statistically significant.

## Results

Of 252 participants who met the inclusion criteria, 215 were included in the analysis after excluding those who were unsure of the wheelchair type used (n = 5) and those whose long-term care claims data and medical claim data were not linked (n =32).

Table 2 presents the characteristics of participants based on wheelchair type. The median age of the participants was 88 years (interquartile range 83-93), and 153 (71.2%) were female. The proportion of participants who used multifunctional wheelchairs before nursing home admission was 44.7% (n = 96 of 215). Multifunctional wheelchair users had a higher care-need level and were more likely to require assistance with sitting, standing up, and transferring than standard wheelchair users. However, no significant differences were observed in the characteristics between the two groups.

**Table 2.** Baseline Characteristics of Participants by Multifunctional and Standard Wheelchair Users.

Participants’ Characteristics	Total (N = 215)	Multifunctional Wheelchair (n = 96)	Standard Wheelchair (n = 119)	p Value*
**Age median (IQR)**	88 (83-93)	87 (84-92)	89 (83-94)	0.261
**Sex (women) n (%)**	153 (71.2%)	68 (70.8%)	85 (71.4%)	0.924
**Care need level n (%)**
Care need level 3	39 (18.1%)	15 (15.6%)	24 (20.2%)	0.314
Care need level 4	121 (56.3%)	54 (56.3%)	67 (56.3%)
Care need level 5	55 (25.6%)	27 (28.1%)	28 (23.5%)
**Comorbidities n (%)**
Dementia	120 (55.8%)	53 (55.2%)	67 (56.3%)	0.872
Cerebrovascular diseases	121 (56.3%)	50 (52.1%)	71 (59.7%)	0.265
Visual deficit	4 (1.9%)	2 (2.1%)	2 (1.7%)	0.828
Depression	8 (3.7%)	4 (4.2%)	4 (3.4%)	0.756
**Medication n (%)**				
Antipsychotics	38 (17.7%)	17 (17.7%)	21 (17.6%)	0.991
Benzodiazepines	47 (21.9%)	17 (17.7%)	30 (25.2%)	0.186
Sedatives hypnotics	49 (22.8%)	20 (20.8%)	29 (24.4%)	0.539
Antihypertensives	136 (63.3%)	54 (56.3%)	82 (68.9%)	0.056
Antidepressants	17 (7.9%)	7 (7.3%)	10 (8.4%)	0.764
**Physical function n (%)**
Sitting
Possible without support	18 (8.4%)	6 (6.3%)	12 (10.1%)	0.510^†^
Possible with self-support	39 (18.1%)	20 (20.8%)	19 (16.0%)
Assistance (partial or full)	96 (44.7%)	47 (49.0%)	49 (41.2%)
Missing	62 (28.8%)	23 (24.0%)	39 (32.8%)
Standing up
Possible without support	0 (0.0%)	0 (0.0%)	0 (0.0%)	0.508^†^
Possible with self-support	48 (22.3%)	21 (21.9%)	27 (22.7%)
Assistance (partial or full)	105 (48.8%)	52 (54.2%)	53 (44.5%)
Missing	62 (28.8%)	23 (24.0%)	39 (32.8%)
Transferring
Independent	7 (3.3%)	1 (1.0%)	6 (5.0%)	0.233^†^
Partial assistance	72 (33.5%)	34 (35.4%)	38 (31.9%)
Full assistance	74 (34.4%)	38 (39.6%)	36 (30.3%)
Missing	62 (28.8%)	23 (24.0%)	39 (32.8%)

IQR: interquartile range. *Age was compared using Student’s t-test; care need level and physical function were compared using the Wilcoxon rank sum test; sex, comorbidities, and medication were compared using the chi-square test. ^†^p Value was calculated after excluding the missing category.

Table 3 provides the number of fractures by type of wheelchair. No fall-related fractures occurred among standard wheelchair users, whereas five fractures were observed among multifunctional wheelchair users.

**Table 3.** Number of Fractures by Multifunctional and Standard Wheelchair Users.

	Multifunctional wheelchair (n = 96)	Standard wheelchair (n = 119)
Fracture n (%)	5 (5.2%)	0 (0.0%)

Table 4 provides information on residents with fall-related fractures. Of the five fractures, four were hip fractures, and one was a forearm fracture. All individuals with fractures used either self-propelled or caregiver-assisted wheelchairs equipped with back supports designed for kyphosis, and one resident used a wheelchair with the aforementioned functions plus a lower seat height than the standard specification. All but one resident required assistance with all three physical functions.

**Table 4.** Fracture Information.

	Care need level	Fracture site	Wheelchair	Physical function		
				Sitting	Standing up	Transferring
Case 1	Care need level 4	Hip fracture	Self-propelled wheelchair/back supports designed for kyphosis/low seat height	Possible with self-support	Possible with self-support	Independently
Case 2	Care need level 4	Hip fracture	Self-propelled wheelchair/back supports designed for kyphosis	Required assistance (partial or full)	Required assistance (partial or full)	Required full assistance
Case 3	Care need level 5	Hip fracture	Caregiver-assisted wheelchair/back supports designed for kyphosis	Required assistance (partial or full)	Required assistance (partial or full)	Required partial assistance
Case 4	Care need level 4	Hip fracture	Self-propelled wheelchair/back supports designed for kyphosis	Required assistance (partial or full)	Required assistance (partial or full)	Required full assistance
Case 5	Care need level 5	Forearm fracture	Caregiver-assisted wheelchair/back supports designed for kyphosis	Required assistance (partial or full)	Required assistance (partial or full)	Required full assistance

## Discussion

To the best of our knowledge, this is the first study to investigate the occurrence of fall-related fractures after nursing home admission by type of wheelchair used before nursing home admission, using administrative claims data in Japan. We identified that approximately 40% of residents who used wheelchair rental services before nursing home admission used multifunctional wheelchairs, and five fractures occurred among residents who used multifunctional wheelchairs, compared with no fractures among those who used standard wheelchairs before nursing home admission. A previous study of geriatric intermediate care facility residents reported that approximately 1% of those who experienced a fall sustained fractures ^(20)^, indicating a high frequency of falls that do not result in fractures. Therefore, although a small number of fractures was observed in our study, a high number of falls may have occurred in the background of the five fractures.

Although no statistically significant differences were observed (Table 2), residents who used multifunctional wheelchairs before nursing home admission tended to have severe care-need levels and required assistance with physical function, compared with residents who used standard wheelchairs. Similarly, residents who experienced fall-related fractures required assistance with physical function (Table 4). Based on these results, the use of multifunctional wheelchairs before nursing home admission might be considered a proxy for lower physical function. Although statistical testing could not be conducted owing to the small number of fall-related fracture cases in this study population, the use of multifunctional wheelchairs before nursing home admission can be considered a predictive factor for fall-related fractures after admission.

Two potential mechanisms may explain the association between pre-admission use of a multifunctional wheelchair and fall-related fractures after nursing home admission. The first mechanism is that residents who used multifunctional wheelchairs before nursing home admission exhibited a high risk of falls, which might have led to fall-related fractures after admission. Residents using multifunctional wheelchairs who experienced fall-related fractures required assistance with physical function and exhibited characteristics such as decreased lower limb strength and impaired balance or trunk functions, which are potential risk factors for falls. Therefore, they might have fallen and experienced fractures due to losing balance when standing or an inability to maintain a sitting posture in a bed or wheelchair.

The second possible mechanism is that residents who used multifunctional wheelchairs before nursing home admission could not use the same kind of multifunctional wheelchairs after admission owing to the lack of wheelchair rental services in nursing homes, which might have led to the fracture-causing falls. A previous study has reported that most of the wheelchairs available in nursing homes are standard models ^(8)^. In addition, another previous study has reported that the most common situation related to falls among wheelchair users requiring assistance with physical function was the buttocks sliding forward on the wheelchair seat ^(20)^, whereas among wheelchair users who were able to perform physical function independently, it was transfer between wheelchairs and beds/toilets ^(20)^. Regarding the functionality of wheelchairs used by the residents who experienced fractures (Table 4), four residents (cases 2, 3, 4, and 5) who required assistance with physical function used wheelchairs equipped with back support designed to accommodate kyphosis (designed to prevent sliding forward), and one resident (case 1) who was independent in physical function used a wheelchair with lower seat height than the standard specification. If the same wheelchairs with these features cannot be used after admission, the residents are at risk of having their buttocks sliding from the wheelchair and are unable to sit deep enough during transfer. These findings, in combination with those of previous studies, indicate that multifunctional wheelchair users who cannot use the same type of multifunctional wheelchairs in nursing homes may susceptible to fall-related fractures.

Although we proposed the previously mentioned mechanisms, whether the latter mechanism―the difficulty to continue using a multifunctional wheelchair after nursing home admission―truly exists remains unclear based on the current results, highlighting the need for further investigations. However, this study sheds light on the issue of the lack of wheelchair rental services in nursing homes, which has not been sufficiently explored.

Our study has several strengths. First, this study used administrative claims data that included all insured persons and all nursing homes in Ibaraki Prefecture rather than a single nursing home, enabling a more comprehensive understanding of the actual situation. Second, the use of long-term care claims data and care-need certification survey data allowed the acquisition of accurate and detailed information about wheelchair types and physical function of nursing home residents.

Nevertheless, the results must be interpreted with caution. First, the actual type of wheelchairs used after nursing home admission was unclear. The residents who used multifunctional wheelchairs before nursing home admission may have difficulty continuing to use the same wheelchair after nursing home admission because multifunctional wheelchairs are rarely available in nursing homes ^(8)^. However, some residents may still have been able to use multifunctional wheelchairs even after admission. Second, the circumstances surrounding fall-related fractures also remain unclear. Although falls among wheelchair users who require assistance or are independent in daily life are often associated with the wheelchair ^(20)^, some fractures may result from unrelated wheelchair incidents, such as falls from a bed or factors involving caregivers during transfers. Third, the types of wheelchairs used after admission and circumstances surrounding fall-related fractures remain unknown. In addition, this was a descriptive study; the causal mechanism, such as whether the fall-related fracture after nursing home admission occurred because of discontinuing the use of multifunctional wheelchairs given the lack of wheelchair rental services in nursing homes, could not be determined. To identify the causal relationship, future studies should investigate the types of wheelchairs used after nursing home admission; circumstances surrounding fall-related fractures; and confounding factors such as disease, medication, limb strength, and balance function. Finally, the generalizability of this study might be limited because it was conducted in a single prefecture, although the data included all older adults within that region. Our results might not be generalizable to other types of long-term care facilities in Japan, such as geriatric intermediate care facilities, or to all nursing homes across Japan.

In conclusion, this study provides foundational preliminary data, emphasizing that residents who used multifunctional wheelchairs before nursing home admission experienced more fall-related fractures after admission than those who used standard wheelchairs before admission. These findings could not clearly reveal the issues related to the lack of wheelchair rental services in nursing homes. However, it sheds light on previously under-researched aspects related to the lack of wheelchair rental services in nursing homes. Future studies should consider adopting prospective designs that can evaluate how the use of multifunctional wheelchairs before nursing home admission leads to fall-related fractures after nursing home admission, using data sources that directly capture the types of wheelchairs used after nursing home admission, as well as the circumstances associated with and causes of fall-related fractures.

## Article Information

### Acknowledgments

We are grateful to the staff members of Ibaraki Prefectural Government for their contributions to data acquisition. We thank Editage (www.editage.com) for its English language editing.

### Author Contributions

Ai Suzuki contributed to the study design, data processing, data analysis, and manuscript drafting. Kazuaki Uda contributed to the study design, supporting the interpretation of the analytical results, and critical revision of the manuscript. Taeko Watanabe contributed to the critical revision of the manuscript. Nanako Tamiya contributed to the study design and critical revision of the manuscript. All authors have read and approved the final manuscript.

### Conflicts of Interest

None

### IRB Approval Code and Name of the Institution

This study was approved by the ethics committee of the Faculty of Medicine, University of Tsukuba (approval number 1595-5).

## References

[1] Annual report on the ageing society [Summary]. FY2024 [Internet]. Cabinet Office Japan. 2024 [cited 2025 Mar 20]. Available from: https://www8.cao.go.jp/kourei/english/annualreport/2024/pdf/2024.pdf

[2] 179th Meeting of the Long-Term Care Insurance Subcommittee of the Social Security Council [Internet]. Ministry of Health Labour and Welfare, Japan. 2020 [cited 2025 Mar 20]. Available from: https://www.mhlw.go.jp/content/12300000/000648154.pdf

[3] Iwagami M, Tamiya N. The long-term care insurance system in Japan: past, present, and future. JMA J. 2019;2(1):67-9. 33681515 10.31662/jmaj.2018-0015 PMC7930803

[4] Clarke P, Chan P, Santaguida PL, et al. The use of mobility devices among institutionalized older adults. J Aging Health. 2009;21(4):611-26. 19282269 10.1177/0898264309333313

[5] Sekikawa S. Wheelchair survey at an aged-care nursing home. In: Ohnabe H, Kubo M, Collins DM, Cooper RA, editors. Selected papers from the Japanese conference on the advancement of assistive and rehabilitation technology. Amsterdam, The Netherlands: IOS Press; 2011. p. 61-6. Japanese.

[6] Sekikawa S. The problem of the wheelchairs at aged-care nursing homes: development of clinical support system and wheelchair for the elderly person to solve this clinical issue. J Jpn Acad Prosthetists Orthotists. 2015;23(1):9-14. Japanese.

[7] The long-term care Insurance System [Internet]. Ministry of Health, Labour and Welfare, Japan. 2024 [cited 2025 Mar 20]. Available from: https://www.mhlw.go.jp/english/topics/elderly/care/2.html

[8] Sotomura A. Nurses’ understanding of the risk factors associated with elderly wheelchair users in facilities covered by public aid long-term care for the elderly. Rounenkangogaku. 2015;20(1):97-104. Japanese.

[9] Report of the Hardware Improvement Subcommittee of the Operation Zero Physical Restraints Promotion Council [Internet]. WAM NET. 2001 [cited 2025 Mar 20]. Available from: https://www.wam.go.jp/wamappl/bb05Kaig.nsf/vAdmPBigcategory20/66966BCD6E98929E49256AA70009EFD7?OpenDocument

[10] Johnell O, Kanis JA, Odén A, et al. Mortality after osteoporotic fractures. Osteoporos Int. 2004;15(1):38-42. 14593451 10.1007/s00198-003-1490-4

[11] Farahmand BY, Michaëlsson K, Ahlbom A, et al. Survival after hip fracture. Osteoporos Int. 2005;16(12):1583-90. 16217590 10.1007/s00198-005-2024-z

[12] Parkkari J, Kannus P, Palvanen M, et al. Majority of hip fractures occur as a result of a fall and impact on the greater trochanter of the femur: a prospective controlled hip fracture study with 206 consecutive patients. Calcif Tissue Int. 1999;65(3):183-7. 10441647 10.1007/s002239900679

[13] Section 4. Human Resource Management. 4.9 Japan’s social security system [Internet]. Japan External Trade Organization. 2024 [cited 2024 Jul 10]. Available from: https://www.jetro.go.jp/en/invest/setting_up/section4/page9.html

[14] Health and medical services [Internet]. Ministry of Health, Labour and Welfare, Japan. 2011 [cited 2025 Mar 20]. Available from: https://www.mhlw.go.jp/english/wp/wp-hw5/dl/23010201e.pdf

[15] Technical Aids information system [Internet]. The Association for Technical Aids. 2024 [cited 2025 Mar 20]. Available from: https://www.techno-aids.or.jp/ServiceWelfareGoodsList.php

[16] Palvanen M, Kannus P, Parkkari J, et al. The injury mechanisms of osteoporotic upper extremity fractures among older adults: a controlled study of 287 consecutive patients and their 108 controls. Osteoporos Int. 2000;11(10):822-31. 11199185 10.1007/s001980070040

[17] Iwagami M, Taniguchi Y, Jin X, et al. Association between recorded medical diagnoses and incidence of long-term care needs certification: a case control study using linked medical and long-term care data in two Japanese cities. Ann Clin Epidemiol. 2019;1(2):56-68.

[18] Donoghue OA, Briggs R, Moriarty F, et al. Association of antidepressants with recurrent, injurious and unexplained falls is not explained by reduced gait speed. Am J Geriatr Psychiatry. 2020;28(3):274-84. 31727515 10.1016/j.jagp.2019.10.004

[19] Woolcott JC, Richardson KJ, Wiens MO, et al. Meta-analysis of the impact of 9 medication classes on falls in elderly persons. Arch Intern Med. 2009;169(21):1952-60. 19933955 10.1001/archinternmed.2009.357

[20] Hasegawa D, Fujita Y, Sakamoto H, et al. Fall occurrence in long-term care facilities―a focus on the movement status of residents. Jpn J Fall Prev. 2016;2(3):23-32. Japanese.

